# Factors associated with HIV infection among delivered women in Sergipe, Brazil

**DOI:** 10.1186/1756-0500-2-156

**Published:** 2009-08-03

**Authors:** Lígia Mara D Lemos, Ricardo Q Gurgel, Juan José L Rivas, Luiz de Souza

**Affiliations:** 1 Federal University of Sergipe, Aracaju, Brazil; 2 Women's Health Programme, Aracaju, Brazil

## Abstract

**Background:**

In Brazil, the number of HIV cases has increased mostly amongst poor less educated women in the northeast region. This combination increased the risk for vertical transmission. This study aims to identify risk factors associated with HIV infection at delivery in Sergipe-NE Brazil.

**Findings:**

This was a case-control study, with 39 cases and 117 controls that gave birth at the official health system hospitals. All patients were tested for HIV at hospital admission, using a rapid test and were interviewed about socioeconomic conditions and health attitudes and practices. Univariate and multivariate logistic analysis were performed to evaluate the factors associated with HIV infection.

In the univariate analysis, association with HIV positivity was found for the variables "antenatal HIV test" (OR: 4.44; CI: 1.93 – 10.29) and "intravenous drug use" (OR = 12.08; 95% CI 1.28 – 8). Three patients were intravenous drug users, all HIV+. After logistic multivariate regression, not being tested for HIV during antenatal care (OR = 4.98; 95% CI: 2.13–12.22; p < 0.001) and lack of knowledge on how to prevent HIV infection (OR = 2.56; 95%CI: 1.09 – 6.27; p = 0.030) were independently associated with HIV positivity.

**Conclusion:**

Drug use, limited knowledge about how to prevent AIDS, and lack of HIV testing during pregnancy were risk factors for infection with HIV. Although it was not conceived to evaluate effectiveness of procedures to prevent vertical transmission, the risk factors here detected may corroborate official recommendation for rapid HIV testing at delivery as an effective procedure to prevent vertical transmission.

## Background

The AIDS epidemic in Brazil shows a downward trend towards stability since 1996, coinciding with the introduction of antiretroviral therapy. The deceleration of the epidemic however did not occur homogeneously within regions. In the Northeast region for example, the incidence rates of AIDS continue to grow [1].

Heterosexual transmission has increased since the early 90s, leading to an increased number of women testing positive for HIV [2,3]. This can be observed by the dramatic change from the 28:1 male-to-female ratio in the beginning of the epidemic to the 1.3:1 ratio of current HIV cases [4]. The number of cases has increased mostly amongst poor women with lower educational levels in the northeast region [1,5].

The increase in the prevalence of HIV infections among females resulted in an increase in the number of children infected via vertical transmission. In Brazil, results from a sentinel surveillance project showed a rate of HIV positivity during pregnancy of 0.413% (95% confidence interval-CI: 0,294–0,533) in 2005 [6]; this is the same as our findings in Sergipe (0.42%) in the same period [7].

In 1997, Brazil adopted a policy of universal HIV testing for pregnant women during antenatal care and at delivery [8], and recommended the use of protocol ACTG 076 (AIDS Clinical Trial Group) [9]. Both testing and medicines are provided free of charge by the national STD/AIDS program. This allowed vertical transmission to be reduced after 1997, although continuing to be a relevant transmission route for HIV in some regions [10].

In Sergipe, sexually transmitted HIV infections are prevalent [4] although there is little information about specific risk factors for infection, especially for pregnant women. Antenatal care is an important opportunity for inclusion of women into public health services. In this state most deliveries (97%) occur inside hospital settings, creating a singular moment to evaluate this group. This study aims to identify the risk factors associated with HIV infection in pregnant women in the public health system (Sistema Único de Saúde – SUS) as a part of a larger project, which introduced the rapid test during pregnancy and delivery in Sergipe State [7].

## Methods

The study was carried out in Sergipe State, located in northeast Brazil, from January 2003 to March 2004. The state has a population of 1,967,791, according to the last census [11], and an average of 37,370 deliveries each year, half of them in the maternity hospitals of Aracaju, the capital city [12]. In Brazil, there are universal free-of-charge antenatal care services and hospital facilities provided for delivery.

This was a case-control study designed to identify risk factors associated with HIV infection in women delivering at three maternity hospitals linked to the official health system. The maternity hospitals were selected because they had the largest number of deliveries in the state. Together they had 58% of all state deliveries in 2003. Two maternity hospitals were located in Aracaju and the other one in Itabaiana, in the central region of Sergipe, 50 km away from Aracaju.

During the study period, all patients (9.215) were tested for HIV before delivery using a rapid test (Determine™ – Abbott Laboratories. Abbott Park, Illinois, U.S.A.). All HIV positive individuals (n = 39) were considered as cases and the next three seronegative patients following them were invited to participate in the study as controls (n = 117). Thirty two percent (n = 2,946) of patients had been tested for HIV during pregnancy; 15 of the 39 positive individuals already knew their seropositivity. There were no refusals to testing or participation. Women undergoing other procedures besides delivery were not included.

The selected patients were interviewed during the first 12 hours after delivery, using a questionnaire based on Ministry of Health forms [SINAN-AIDS (disease notification form) and SI-CTA (Counselling and testing form)] available in . Information was analyzed dicotomically and grouped into socio-demographic, risk attitudes and practices, and obstetric and antenatal care information.

Variables included in analysis were: age (< 19 years old or ≥ 19 years old), years of schooling (≤ 4 years or > 4 years), occupation (employed and not employed), marital status (stable union or single/divorced), and income (with income or without income), last year's number of partners (1 or > 1), intravenous drug use (yes or no). The knowledge about how to prevent AIDS was obtained using three open questions routinely used at Sergipe Health Department AIDS Programme (have you heard about AIDS? Do you know how to prevent? Do you think you were at risk to be infected with HIV? Why?). These questions were recoded as "right" or "wrong" according to a list of situations previously considered as "right or wrong", according to the literature.

Antenatal care attendance was evaluated with the following questions: "had antenatal care"; "do you have your antenatal care records?"; "Was submitted to HIV testing during pregnancy?"

For statistical analysis, variables were tested using the Fisher exact test to evaluate each variable's independent association with the variable "positive HIV test" (case versus controls). Those with a p value < 0.25 were selected for the exact multiple logistic regression (occupation, knowledge about HIV prevention, income, antenatal care, antenatal HIV test, marital status, age, schooling).

The logistic regression strategy was used as recommended by Hosmer & Lemeshow [13]. We used stepwise forward procedure and significance was established as α < 0.05. We used the software SAS 9.1 to analyze the data.

The Ethics Committees of the Federal University of Sergipe and Conceição Hospital Group in Porto Alegre approved the study. All participants signed a consent form prior to HIV testing and questionnaire application.

## Results

Both groups came from low socioeconomic strata; aged predominantly > 19 years; half were living in the interior and married. They also had a large percentage of unemployment and low schooling, but attended, in a high proportion, to antenatal care services (82 and 93%) (Table 1). Association with HIV **positivity **was found **for **the variables "antenatal HIV test" (OR: 4.44; CI: 1.93 – 10.29) and "intravenous drug use" (OR = 12.08; 95% CI 1.28 – ∞). Three patients only were intravenous (IV) drug users, all of them HIV positive. For this reason, this variable was included for the univariate analysis only; this estimate was considered biased and imprecise. This group did not attend to antenatal visits, and two **women **did not know how to prevent AIDS, although both had more than 4 years of schooling.

**Table 1:** Socioeconomic factors, attitudes, and antenatal care use in delivered women with and without an HIV positive test, Aracaju-2005

Variables	cases		controls				
	n	%	n	%	*OR	*IC	p**
**Occupation**							**0.094**
Employed	20	51.3	42	35.9	1.88	0.84 – 4.17	
Unemployed	19	48.7	75	64.1			

**Marital status**							**0.164**
Without partner	16	41.0	33	28.2	1.77	0.77 – 4.00	
With partner	23	59.0	84	71.8			

**Age**							**0.161**
≥19 years	37	94.6	100	85.5	3.14	0.69 – 29.41	
<19 years	2	5.1	17	14.5			

**Knowledge about HIV prevention**							**0.062**
No	22	56.4	45	38.5	2.07	0.93 – 4.63	
Yes	17	43.6	72	61.5			

**Schooling**							**0.238**
≤ 4 years	16	41.0	35	29.9	1.63	0.71 – 3.67	
> 4 years	23	59.0	82	70.1			

**Income**							**0.140**
Without income	15	38.5	62	53.0	1.80	0.81 – 4.08	
With income	24	61.5	55	47.0			

**Number of partners**							**0.332**
1	39	100.0	112	95.7	2.30	0.31 – ∞	
>1	0	0	5	4.3			

**Intravenous drug use**							**0.015**
No	36	92.3	117	100.0	12.08	1.28 – ∞	
Yes	3	7.7	0	0			

**Antenatal care**							**0.058**
No	7	17.9	8	6.8	2.98	0.84 – 10.15	
Yes	32	82.1	109	93.2			

**Antenatal HIV test**							**<0.001**
No	24	61.5	31	26.5	4.44	1.93 – 10.29	
Yes	15	38.5	86	73.5			

Total	39		117				

* Reference: 2^nd ^category

Exact Logistic Regression

** Fisher exact test

After logistic multivariate regression, not being tested for HIV during antenatal care (OR = 4.98; 95% CI: 2.13–12.22; p < 0.001) and lack of knowledge on how to prevent HIV infection (OR = 2.56; 95%CI: 1.09 – 6.27; p = 0.030) were independently associated with HIV positivity. All other variables, when adjusted to the variable "antenatal HIV test" and "knowledge about HIV prevention," were not significant. HIV positive testing at delivery occurred five times more frequently among deliveries that did not have an antenatal HIV test than among deliveries with antenatal HIV testing. "Do not know how to prevent AIDS" had a two times increased risk of being seropositive. The variable "antenatal care," almost significant at univariate analysis (p = 0.058), did not confirm significance after regression (p = 0.268).

A particular group of 10 cases (data not shown), who reported having had sexual intercourse without protection with partners known as seropositive, had a positive HIV test. Half of them were employed, married, and had more than 4 years of schooling; all of them were 19 years of age or older and had a single partner; they did not use drugs and denied having sex for money; they did not keep their antenatal visit records and only one attended regularly to the antenatal visits; two of them were tested for HIV during pregnancy. No controls had this condition.

## Discussion

The present study showed that knowledge about how to prevent AIDS, and effectively have HIV testing during pregnancy are related to a lower seropositivity for HIV at delivery. This reinforces the importance of information for HIV prevention. Limited access to antenatal care was more frequent in cases, and is in accordance with other studies where there are several situations in which health services are not effective to enrol and keep at-risk patients in regular follow up [14,15]. In some places and situations, pregnancy is the only opportunity for some women to access the health system [14]. The starting point for preventing HIV vertical transmission is better access to the public health system and consequently a serologic screening test for pregnant women as early as the pregnancy is detected [16-18].

The control group showed better knowledge about how to prevent AIDS. The link between information and perception about HIV transmission risk is not always detected [19], and this may be due to health professionals' inadequate efforts [15,20]. Improving the level of information in pregnant women could help to reduce maternal and child morbidity and mortality [21,22].

Special attention must be paid to the existence of two adolescent seropositive participants in our study. This is considered an especially vulnerable group, in need of particular strategies to increase their knowledge about HIV transmission and different approaches to effectively prevent disease transmission [23]. A high percentage of adolescents reported at least one risky sexual experience. Information quality was considered as inadequate and friends were referred as the main source of information about HIV and AIDS [24].

Behavioral factors play an important role and deserve close analysis, as we detected ten patients infected by partners they already knew to be HIV seropositive. The wish to have their own children and the confidence that emerges between stable partners results in less use of condoms, increasing women's vulnerability [3,25,26]. Uneven gender relations and men's unfaithfulness are considered important factors for HIV transmission among women [27,28]. It is noteworthy that all these participants declared to have a single partner in the last year, and all the partners were infected with HIV.

## Conclusion

Drug use, limited knowledge about how to prevent AIDS, and lack of HIV testing during pregnancy were considered risk factors for infection with HIV. Although this study was not conceived to evaluate effectiveness of procedures to prevent vertical transmission, the risk factors here detected may corroborate the Ministry of Health recommendation for rapid HIV testing at delivery as an effective procedure to prevent vertical transmission.

## Competing interests

The authors declare that they have no competing interests.

## Authors' contributions

LMDL conducted the study as part of a Master of Public Health degree, supervised the research project, and provided expertise in survey instruments, development and data analysis, and manuscript preparation. RQG supervised the research project and provided expertise in survey instruments, development and data analysis, and manuscript preparation. JJLR assisted with data collection and manuscript preparation. LS performed statistical analysis and assisted with manuscript preparation. All authors read and approved the revised final manuscript.
